# Small-pox in India

**Published:** 1865-05

**Authors:** 


*Small-pox in India.*—Small-pox is ravaging Lahore; 7000 out of a population of 50,000 of the native inhabitants having fallen victims to it within two months.

